# Assessing the Relationship between Vitamin D_3_ and Stratum Corneum Hydration for the Treatment of Xerotic Skin

**DOI:** 10.3390/nu4091213

**Published:** 2012-09-04

**Authors:** Meghan Russell

**Affiliations:** Johnson and Johnson Skin Research Center, CPPW, Johnson & Johnson Consumer Companies, Inc., Skillman, NJ 08558, USA; Email: mrusse2@its.jnj.com; Tel.: +1-908-874-1259; Fax: +1-908-874-1254

**Keywords:** vitamin D, cholecalciferol, skin moisturization, wintertime dry skin

## Abstract

Vitamin D_3_ has been called the “sunshine” vitamin since the formation of vitamin D is mediated by exposure to sunlight. Vitamin D_3_ is linked to many health benefits, however serum levels of vitamin D_3_ have been decreasing over the last few decades and the lower levels of vitamin D_3_ may have consequences on normal physiology. We investigated the association between serum 25-hydroxyvitamin D (25(OH)D) levels and stratum corneum conductance as well as the effect of topical application of cholecalciferol (vitamin D_3_) on dry skin. Eighty three subjects were recruited and blood serum levels and skin conductance measurements were taken after a one week washout. A correlation was observed between vitamin D levels and skin moisture content, individuals with lower levels of vitamin D had lower average skin moisture. Subsequently, a 3-week split leg, randomized, vehicle controlled clinical study was conducted on a subset of 61 of the above individuals who were identified with non-sufficient vitamin D serum levels. Topical supplementation with cholecalciferol significantly increased measurements of skin moisturization and resulted in improvements in subjective clinical grading of dry skin. Taken together our finding suggest a relationship between serum vitamin D_3_ (25(OH)D) levels and hydration of the stratum corneum and further demonstrate the skin moisture benefit from topical application of vitamin D_3_.

## 1. Introduction

Vitamin D has long been known to increase calcium absorption for bone health, but recently has been associated with several other health benefits including cancer prevention, autoimmune disease and cardiovascular disease [1]. Vitamin D_3_ is synthesized in the skin and lasts twice as long as vitamin D ingested from the diet [2]. 7-Dehydrocholesterol is converted to cholecalciferol (vitamin D_3_) in the skin when exposed to UVB radiation and then hydroxyalated to 25 hydroxyvitamin D_3_ (25(OH)D) and then further hydroxyalated to 1,25 dihyroxyvitamin D_3_ (calcitriol). Calcitriol is known to modulate proliferation and differentiation of keratinocytes [3] and analogs of vitamin D_3_ have been used to normalize hyperproliferation present in psoriatic skin. Research has been conducted on serum vitamin D_3_ (25(OH)D) levels, but little is known about skin levels of vitamin D_3_ and even less is known about its topical benefits in normal skin. There has been research showing that vitamin D serum deficiency could be related to severity of atopic dermatitis, as skin barrier dysfunction [4]. This investigation was conducted to examine the association between serum 25-hydroxyvitamin D (25(OH)D) levels and stratum corneum hydration levels based on conductance measurements. We further explored the effect of topical application of cholecalciferol (vitamin D_3_) on dry skin. 

## 2. Methods

### 2.1. Baseline Study

A total of 83 female subjects (age 18–45, *n* = 51 Caucasian (Fitzpatrick skin type I–IV) and *n* = 32 African Americans (Fitzpatrick skin type V–VI)) were recruited for this study which was conducted at an independent clinical site in the northeast US during March 2010. Subjects below the age of 45 were selected with no symptoms of menopause or pre-menopause as not to confound the results. Recruitment was designed to select for subjects with lower serum vitamin D_3_ levels, since addition of topical vitamin D_3_ might be most beneficial for this population. Inclusion criteria included a lifestyle with self-perceived poor eating habits, including less than 227 g of milk consumption per day and no vitamin supplementation. Subjects were excluded if they had vacationed in a sunny climate or had frequented tanning beds in the last three months as well as overly active in outside activities. Subjects were also excluded if they had active symptoms of skin conditions on their legs, including atopic dermatitis. Included were females who use moisturizer on their legs at least five times per week based upon the target audience for the finished product. The clinical investigation, including the informed consent, was reviewed by an Institutional Review Board (Allendale, NJ) and approved prior to the start of the study.

A blood sample was taken from subjects at baseline for serum vitamin D_3_ (25(OH)D) determination via HPLC/MS (Vitamin Diagnostics Laboratory in Keyport, NJ). 

Subjects had a one week washout where they discontinued all moisturizer use and substituted their body cleanser with a supplied body wash (Johnson’s^®^ head-to-toe^®^ baby wash) known to be mild to be consistent across all panelists. Skin conductance measurements were taken in triplicate on the lower lateral leg at the end of the one week period with a Skicon 200 (I.B.S. Co. Ltd., Japan) using a MT-8C probe and mean values were calculated.

### 2.2. Treatment Study

Subsequently, a 3-week split leg, randomized, vehicle controlled clinical study was also conducted on a subset of 61 of the above individuals (*n* = 34 Caucasians (Fitzpatrick skin type I–IV) and *n* = 27 African Americans (Fitzpatrick skin type V–VI)) who were willing to continue in the study and were in the non-sufficient category (insufficient and deficient categories). Subjects applied moisturizer containing 10 μg/g vitamin D_3_ (cholecalciferol) and a vehicle twice a day on each leg. The vehicle used in both groups was an oil in water emulsion that contained glycerin, petrolatum and dimethicone. The application was self-performed with instructions of two pumps per lower leg from the knee to the ankle. The leg is a well characterized body location for conducting studies of skin moisture content and for evaluating the appearance of dry skin. The vehicle was identical to the active formula just without containing cholecalciferol. At baseline and 3 weeks skin conductance, visual evaluation of dryness by an expert grader and self-assessment were performed. The expert grader measured on a predetermined scale of 0–4 for normal to severe skin based on previous training. The subjects used a 0–10 scale with descriptive anchors to self report skin conditions. Both the expert grader and subjects were blinded to the active and the lotions appeared identical in the same packaging. All measurements were performed in a humidity and temperature controlled room with no current product application on their legs.

### 2.3. Statistical Analysis

ANOVA was used to compare multiple independent groups for deficient *vs.* insufficient *vs.* sufficient. The Wilcoxon Signed Rank test was used to compare treatment *vs.* placebo for the same person. 

## 3. Results

**Figure 1:**
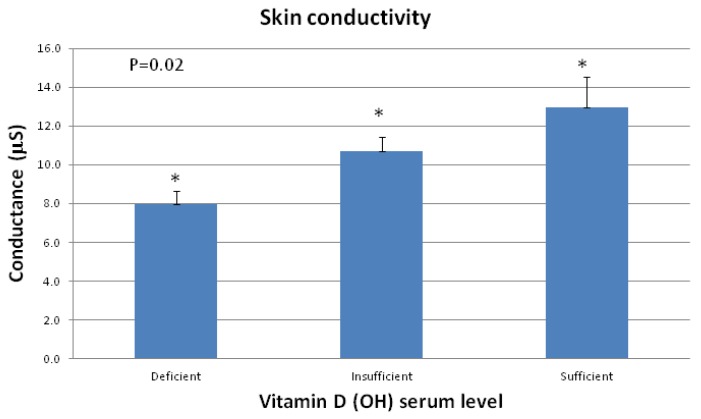
Subjects were categorized by their vitamin D (OH) serum levels. A statistically significant (ANOVA, *p* < 0.05) effect of serum 25(OH)D levels and skin conductance was observed. As serum 25(OH)D levels increased, skin conductance also increased.

Serum 25(OH)D levels ranged from 4.8 to 48.8 ng/mL with 19 subjects classified as deficient (<10 ng/mL), 53 as insufficient (10–30 ng/mL) and 11 subjects as sufficient (>30 ng/mL) based on published literature [5]. A non-invasive measurement was used to assess stratum corneum water content. This instrument measures the high frequency conductance of skin which correlates with skin surface water content [6]. Figure 1 shows that as serum 25(OH)D levels increased, skin conductance also increased (*p* = 0.02, ANOVA). Mean ± SEM values were 7.9 ± 0.69 μS, 10.7 ± 0.72 μS and 13.0 ± 1.57 μS for deficient, insufficient and sufficient subjects respectively.

**Figure 2:**
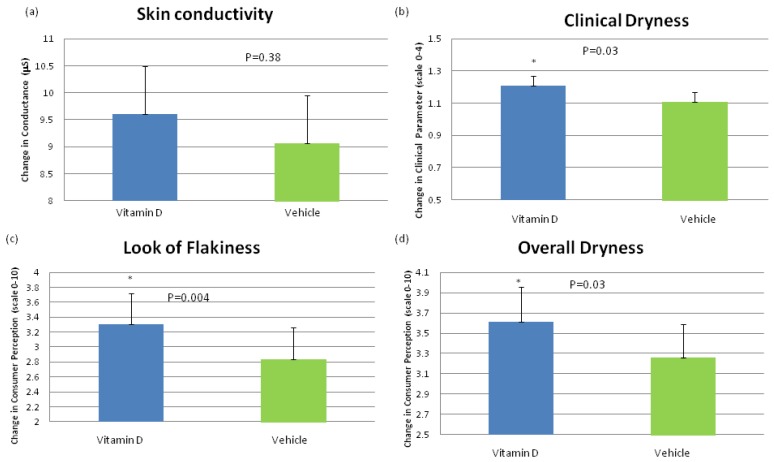
Mean change at week 3 from baseline for (**a**) skin conductivity; (**b**) clinical grading of visual dryness; (**c**) subject perception of visual skin dryness; and (**d**) subject perception of overall dryness. A subset of non-sufficient-vitamin D (OH) serum subjects was analyzed. The addition of vitamin D to a moisturizing formula significantly (Wilcoxon Signed Rank Test, *p* < 0.05) improved clinical grading of visual dryness and consumer perception of flakiness and dryness over the moisturizing formula alone. There was a trend in vitamin D over the vehicle in skin conductivity.

In the subset of subjects with non-sufficient vitamin D_3_ levels, (*n* = 61 including those deficient and insufficient), the addition of vitamin D_3_ to the moisturizing formulation significantly improved (*p* < 0.05 Wilcoxon Signed Rank Test) dryness both in clinical grading and by self assessment over the vehicle at 3 weeks (Figure 2b,d). The look of flakes was also statistically significant (*p* < 0.05 Wilcoxon Signed Rank Test) by self assessment (Figure 2c). Vitamin D_3_ addition to a topical moisturizer increased skin conductance over the vehicle, but did not reach statistical significance in this study utilizing ANOVA (Figure 2a). There was a significant increase in conductance when comparing before and after treatment in both groups. This is probably due to the high emolliency of the vehicle used.

## 4. Discussion

The number of patients with non-sufficient vitamin D_3_ levels may be rising in the population [7]. This could be due to the low consumption of vitamin D fortified foods and the increased awareness of skin cancer through UVB exposure [1]. There are a few demographic groups that are more at risk for being vitamin D_3_ (25(OH)D) insufficient including females, African Americans, and those with higher body mass index [8]. It is estimated that 3 out of every 4 Americans [9] and 1 billion people worldwide are insufficient [1]. 

The current study demonstrates a relationship between a subject’s vitamin D_3_ (25(OH)D) serum levels and their innate skin hydration state. These results in Figure 1 demonstrate that as vitamin D_3_ serum level decreases, skin conductance measurements also decreased indicating that the subject’s skin was drier. Furthermore, topical application of vitamin D_3_ was shown to enhance skin conductance reflected in an increase in moisture levels in the skin for subjects with non-sufficient levels of vitamin D_3_. Interestingly, there is a strong seasonal variation in 25-hydroxyvitamin D levels with significantly lower values in the winter which coincides with wintertime dry skin [10]. The onset of winter xerosis results in a transient but acute perturbation of filaggrin proteolysis [11] and vitamin D analogs have been shown to increase filaggrin expression [12]. Vitamin D_3_ (cholecalciferol) may have potential role in increasing filaggrin and NMF. These results suggest that diminished 25-hydroxyvitamin D levels may be a factor contributing to xerotic skin. Other research supports the hypothesis that vitamin D_3_ serum deficiency may be related to dry skin states including atopic dermatitis [4]. 

## 5. Conclusions

Overall these findings suggest a relationship between serum vitamin D_3_ (25(OH)D) levels and hydration of the stratum corneum and further demonstrate the skin moisture benefit from topical application of vitamin D_3_. Future studies could explore optimal levels of skin vitamin D and the relationship with serum levels, as well as mechanistic studies to understand the influence of vitamin D_3_ on xerotic skin.
